# Automatic Processing of Emotional Words in the Absence of Awareness: The Critical Role of P2

**DOI:** 10.3389/fpsyg.2017.00592

**Published:** 2017-04-20

**Authors:** Yi Lei, Haoran Dou, Qingming Liu, Wenhai Zhang, Zhonglu Zhang, Hong Li

**Affiliations:** ^1^College of Psychology and Sociology, Shenzhen UniversityShenzhen, China; ^2^Research Center for Brain and Cognitive Neuroscience, Liaoning Normal UniversityDalian, China; ^3^School of Psychology, Nanjing Normal UniversityNanjing, China; ^4^College of Education Science, Chengdu UniversityChengdu, China

**Keywords:** emotional word, continuous flash suppression, P2, unconscious, semantic processing

## Abstract

It has been long debated to what extent emotional words can be processed in the absence of awareness. Behavioral studies have shown that the meaning of emotional words can be accessed even without any awareness. However, functional magnetic resonance imaging studies have revealed that emotional words that are unconsciously presented do not activate the brain regions involved in semantic or emotional processing. To clarify this point, we used continuous flash suppression (CFS) and event-related potential (ERP) techniques to distinguish between semantic and emotional processing. In CFS, we successively flashed some Mondrian-style images into one participant's eye steadily, which suppressed the images projected to the other eye. Negative, neutral, and scrambled words were presented to 16 healthy participants for 500 ms. Whenever the participants saw the stimuli—in both visible and invisible conditions—they pressed specific keyboard buttons. Behavioral data revealed that there was no difference in reaction time to negative words and to neutral words in the invisible condition, although negative words were processed faster than neutral words in the visible condition. The ERP results showed that negative words elicited a larger P2 amplitude in the invisible condition than in the visible condition. The P2 component was enhanced for the neutral words compared with the scrambled words in the visible condition; however, the scrambled words elicited larger P2 amplitudes than the neutral words in the invisible condition. These results suggest that the emotional processing of words is more sensitive than semantic processing in the conscious condition. Semantic processing was found to be attenuated in the absence of awareness. Our findings indicate that P2 plays an important role in the unconscious processing of emotional words, which highlights the fact that emotional processing may be automatic and prioritized compared with semantic processing in the absence of awareness.

## Introduction

Emotional words hold an important place in social communication in the modern world. When we see an emotional word, it transmits two main kinds of information. One is semantic information, which contains the meaning of the word, which activates the left lateral occipitotemporal sulci (Dehaene and Cohen, 2011), inferior frontal gyrus (Mestres-Missé et al., 2008; Chou et al., 2012), and angular gyrus (Horwitz et al., 1998; Seghier, 2013); the other is emotional information, which includes the biological value or social significance (Fox et al., 2001), which activates the amygdala (Isenberg et al., 1999; Garavan et al., 2001; Tabert et al., 2001; Hamann and Mao, 2002; Compton et al., 2003; Kensinger and Schacter, 2006; Herbert et al., 2009; García-García et al., 2016), orbitofrontal gyrus and bilateral inferior frontal gyrus (Nakic et al., 2006), anterior cingulate gyrus (Posner et al., 2009), and lingual gyrus (Kuchinke et al., 2005). Although semantic and emotional information processing in the brain may largely overlap (Duncan and Barrett, 2007; Shackman et al., 2011; Raz et al., 2012, 2014), these results suggest that semantic and emotional processing of words activated different brain regions during a visible condition. However, it remains unclear to what extent the emotional and semantic processing of words can take place in the absence of conscious awareness.

In the invisible condition, to render the word stimuli invisible, we adopted an effective paradigm called continuous flash suppression (CFS). This interocular suppression technique has been known as a pivotal tool for exploring the visual awareness (Lin and He, 2009; Eo et al., 2016). In the CFS paradigm, some Mondrian-style images flash successively into the dominant eye steadily, which suppresses the experimental materials projected to the non-dominant eye (Kim and Blake, 2005; Tsuchiya and Koch, 2005).

At present, there is no consensus about unconscious processing of words, regardless of unconscious semantic processing or subliminal emotional processing. Firstly, there are conflicting findings for unconscious semantic processing. Some studies have reported the semantic processing of word stimuli in the unconscious conditions (Dehaene et al., 2006; Jiang et al., 2007; Wang and Yuan, 2008; Sklar et al., 2012; Ortells et al., 2016). However, other studies have provided the opposite evidence that the semantic processing of verbal stimuli cannot occur when they are rendered invisibly (Zimba and Blake, 1983; Kang et al., 2011; Heyman and Moors, 2014; Hesselmann et al., 2015). Secondly, the subliminal emotional processing of words remains questionable. For example, in a behavioral study, Sklar et al. (2012) reported that the negative word expression break suppression faster than the neutral word expression, while Yang and Yeh (2011) showed that the negative words took more time to break suppression than the neutral words. Using fMRI, Ortigue et al. (2007) showed that the masked lovers' names activated the fusiform and angular gyri, indicating that emotion-loaded word can activate the emotion-related and word-related areas. In contrast, Hoffmann et al. (2015) found that there was no significant difference of the relevant brain regions between masked emotional words and neutral words in the invisible condition. These fMRI results are inconsistent partly due to the fact that this technique has a low temporal resolution, which does not provide the temporal course for emotional word processing.

Due to high temporal resolution, ERPs are able to reflect the temporal processing of emotional stimuli. We focused on the P2 component for the following reasons. First, P2 was related to attention and categorization around 150–300 ms post-stimulus onset (Antal et al., 2001; Crowley and Colrain, 2004). Second, a plethora of research has found that emotional effect takes place in the P2 time-window in visible conditions (Begleiter and Platz, 1969; Begleiter et al., 1979; Williamson et al., 1991; Schapkin et al., 2000; Ortigue et al., 2004; Kanske and Kotz, 2007; Wang and Bastiaansen, 2014). For instance, Herbert et al. (2006) observed that negative words elicited larger P2 component compared with neutral words. Finally, at the time window around 250 ms after stimulus onset, there exists a positive-going wave (P250) at the whole brain, which is related to both automatic semantic processing and early word recognition (García-Larrea et al., 1992; Kim et al., 2001; Hill et al., 2002; Liu et al., 2009). For example, Chung et al. (2010) have found the P250 reflects that semantic memory network is activated in the semantic processing of Chinese words.

Until now, few studies have distinguished the two types of processing simultaneously in one experiment in absence of conscious awareness (Yang and Yeh, 2011). In the current study, we used an electroencephalography (EEG) method to clarify the extent to which the semantic and emotional processing of words can occur in the absence of awareness within the time-window of P2. The participants observed negative, neutral, and scrambled word stimuli during the visible condition and the invisible condition. In order to distinguish the semantic and emotional processing, we adopted a kind of scrambled stimuli that removed the semantic information and preserved the spatial location features (Yang and Yeh, 2011). We compared scrambled words with neutral words as the semantic process and also compared negative words with neutral words as the emotional process. Based on previous results (Bernat et al., 2001; Herbert et al., 2006; Kang et al., 2011), we predict that emotional processing of words would occur even when the stimuli were presented unconsciously, but semantic processing would be suppressed in absence of awareness.

## Materials and methods

### Participants

Sixteen right-handed students (10 female; mean age 22.9 years) from Liaoning Normal University participated in the experiment and were compensated $4.50 after the experiment. They were all native Chinese speakers and had normal or corrected-to-normal visual acuity with no psychiatric or neurological history. All participants provided written informed consent. The protocol was approved by the Ethics Committee of Liaoning Normal University.

### Stimuli

We used 30 negative Chinese two-character words (e.g., ”悲伤,” sadness) and an equal number of neutral words (e.g., ”规则,” rule), which were selected from the Chinese Affective Words System (CAWS; Luo and Wang, 2004; Yi et al., 2015). All of the words were nouns (see Supplementary Material). Negative words and neutral words differed significantly in valence [mean: negative = 2.72, neutral = 5.11; *t*_(58)_ = −34.79, *p* < 0.001] and arousal [mean: negative = 5.80, neutral = 4.54; *t*_(58)_ = 5.75, *p* < 0.001]. However, they were matched in stroke numbers [mean: negative = 16.77, neutral = 16.37; *t*_(58)_ = 0.362, *p* = 0.719], word frequency [mean frequency in 15 million words, negative = 0.002887%, neutral = 0.002357%; *t*_(58)_ = −0.562, *p* = 0.578; http://www.cncorpus.org/] and concreteness (this dimension rating was obtained through the use of a seven-point scale by 27 new participants [−3 to 3: very abstract to very concrete; mean: negative 0.72, neutral 1.07; *t*_(58)_ = −1.154; *p* = 0.253]). The scrambled word stimuli were constructed by dividing the negative and the neutral words into 5 × 10 blocks and then arranging them randomly. Separating the words removed the semantic information and preserved the effects of spatial location.

To create the interocular suppression, Mondrian images, and stimuli were projected onto each eye of the participant through a four-mirror stereoscope that included two intermediate mirrors (angled ± 45° orthogonally) between two adjustable mirrors. Mondrian patches (extended 7.16° × 10.03°, visual angle) were generated using Matlab 7.0 software and filled an outer frame (visual angle 8.60° × 11.36°) with the colors black and white. The stimuli were drawn using black characters at 28.6% contrast (the contrast of the stimuli was defined as the luminance difference between the background and the luminance of the words divided by the luminance of the background; Tsuchiya and Koch, 2005; Kang et al., 2011). The screen luminance of the word stimuli was set at 20% of the maximal screen luminance (dark gray), and the screen luminance of the background was set at 28% (light gray). Stimuli were presented on a 48 cm CRT (cathode ray tube) monitor (1024 × 768 resolution at 100 Hz frame rate) and controlled by E-Prime 2.0 software. Each participant was seated 60 cm in front of the computer screen with their head on a chin-rest, and responded by using a keyboard.

### Design and procedure

**Figure 1:**
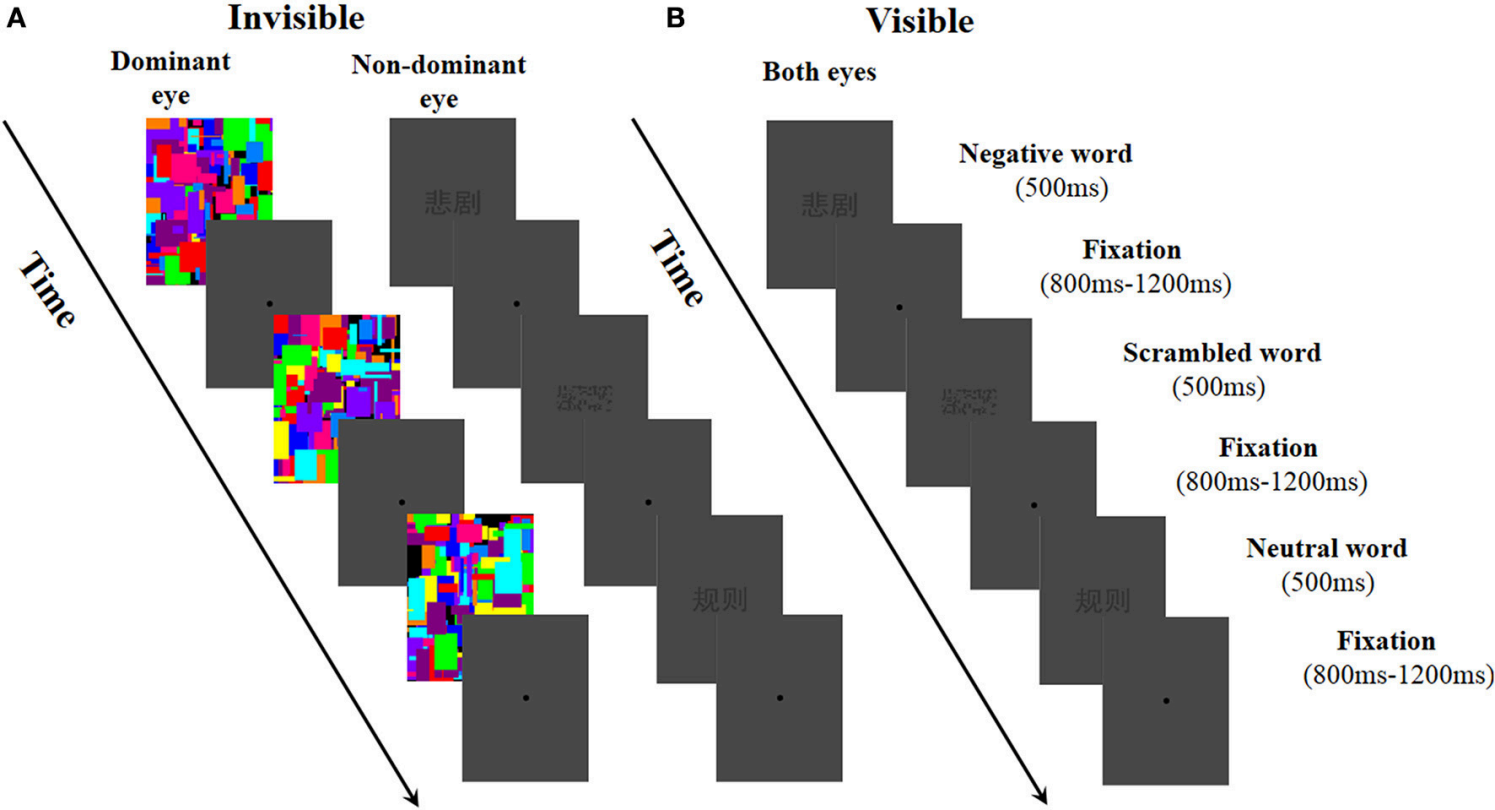
**Experimental procedure used in this study. (A)** In the invisible condition, Mondrian-style images were presented to the dominant eye at a rate of 20 Hz while negative, neutral, and scrambled words were randomly presented to the non-dominant eye. **(B)** In the visible condition, negative, neutral, and scrambled words were randomly presented to both of the participant's eyes.

We used a within-subject experimental design in which two factors—word type (negative, neutral, and scrambled words) and awareness states (visible and invisible light conditions)—were manipulated. To control participants' fatigue, neutral, negative, and scrambled word stimuli were presented randomly within one block consisting of 108 trials in the visible condition. With the invisible condition, each block included 60 trials. Each observer took part in five visible blocks and nine invisible blocks of words. The participants could take a rest between the blocks. The total experimental session lasted ~50 min. To assess their eye-dominance, the participants were asked to view an object through a hole made by their own fingers (the “Miles test”; Mendola and Conner, 2007; Axelrod et al., 2014). In the invisible condition, to make the word stimuli invisible, the Mondrian images were changed at a rate of 20 Hz (50 ms per image) and projected onto the dominant eye of each participant. At the same time, the negative, neutral, and scrambled words were presented to the non-dominant eye. At the beginning of each trial, the word stimuli and Mondrian images (extended 3.15° × 1.43°) were presented for 500 ms, and then followed by an 800–1200 ms randomized fixation (a dot, *d* = 0.38°) in an outer frame, representing an inter-trial interval (ITI; see Figure 1A). In the visible condition, in order to make the participants aware of the word stimuli, we replaced the Mondrian images that had been presented to the dominant eye with word stimuli. The other parts of the visible condition remained the same with the invisible condition (see Figure 1B).

In both the invisible and visible condition, the participants were asked to pay attention to the stimuli and complete a feature detection task. If the participants saw the word stimuli (negative, neutral, and scrambled words), they were instructed to press the “f” key; if not, they pressed the “j” key. The buttons “f” and “j,” which were pressed by the right and left hands—associated with seen and unseen—were counterbalanced across participants. The sequences of the two conditions (invisible and visible) were also counterbalanced across participants. After completing the experiment, the participants were asked to recall the experience of each word when confronted with these words in the formal experiment, and rate the valences and arousals relating to each word using a 9-point Likert scale (valence 1–9; arousal 1–9). The results showed that an emotional word could effectively evoke an affective experience. The valence of the negative words (*M* ± *SE*, 3.28 ± 2821) were significantly lower than those of the neutral words (4.90 ± 0.11; *t* = −7.01, *df* = 30, *p* < 0.001). Moreover, the arousal induced by the negative words (5.26 ± 0.35) was much higher than that induced by the neutral words (3.60 ± 0.33, *t* = 3.451, *df* = 30, *p* = 0.002). Next, the participants completed a subjective report on whether the words presented in the experiment could be seen or not. All the participants reported that they did not see the word stimuli in the invisible condition but did distinguish the negative, neutral, and scrambled words in the visible condition. Moreover, the results of the feature detection task revealed that the participants reported not seeing any words in 99.89% of the trials.

### Electrophysiological recording and analysis

Electroencephalographic (EEG) data were recorded from 64 electrodes using the Active Two system (BioSemi, the Netherlands). These electrodes were referenced online to averaged right and left mastoids. A horizontal electrooculogram (EOG) was recorded from an electrode placed 1.5 cm from the external canthus of the right eye. A vertical EOG was recorded from an electrode placed 1.5 cm below the left eye. The signal was recorded at a sampling rate of 512 Hz, filtered at 0–104 Hz and stored for offline analysis. EEG data were edited with NeuroScan equipment (Compumedics) after data conversion using PolyRex software (as described by Graux et al., 2014). Semiautomatic correction of eye movements was applied using an ICA filter transform developed by Brain Products (BP, Germany). A 0.1–30 Hz IIR bandpass filter was applied (24-dB/oct slope). The ERP waveforms were time-locked to the onset of the stimulus and corrected with a −100 to 0 ms baseline before the stimuli were applied. The remaining artifacts with amplitudes higher or lower than +80 μV were automatically rejected.

P2 was investigated in a time-window of 220–280 ms and was calculated as mean amplitude (Chung et al., 2010; Liu et al., 2013). We defined a mid-line ROI to test the P2 component (from five mid-line electrode sites: Fz, FCz, Cz, CPz, and Pz; Yun et al., 2011). Two-way repeated measures ANOVA was used to analyze the ERPs with awareness states and word types as within-subject factors. Emotional effect was calculated by the differences in the P2 waves between the negative words and the neutral words. Semantic effect was calculated by the differences in the P2 waves between the neutral words and the scrambled words. We also used two-way repeated measures ANOVA to analyze the differences between waves, with awareness states and effects (emotional effect or semantic effect) as within-subject factors. When Mauchly's test indicated that the assumption of sphericity had been violated, Greenhouse–Geisser correction would be used to correct the degree of freedom. Bonferroni correction was used to adjust the *p*-value for all pairwise comparisons.

## Results

### Behavioral data

We rejected the outliers of reaction time (RT) that were outside the range of ±2.5 standard deviations from the mean (<1%) and error trials (mean error rate = 1.38%). The results showed that the interaction of word type and awareness state was not significant [*F*_(2, 30)_ = 2.679, *p* = 0.119, η_*p*_^2^ = 0.152]. However, the main effect of awareness state was found to be significant [*F*_(1, 15)_ = 20.480, *p* < 0.001, η_*p*_^2^ = 0.563]. The main effect of word type was also significant [*F*_(2, 30)_ = 7.354, *p* = 0.013, η_*p*_^2^ = 0.329]. The simple effect of word type in the visible condition [*F*_(2, 30)_ = 4.656, *p* = 0.017, η_*p*_^2^ = 0.237] revealed that negative words (mean RT, 394.169 ms) were faster than the scrambled words (435.437 ms) and neutral words (401.817 ms). The simple effect of word type in the invisible condition [*F*_(2, 30)_ = 1.494, *p* = 0.241, η_*p*_^2^ = 0.091, mean RT: negative word = 689.858 ms, neutral word = 691.949 ms, scrambled word = 695.799 ms] was not significant.

### P2

**Figure 2:**
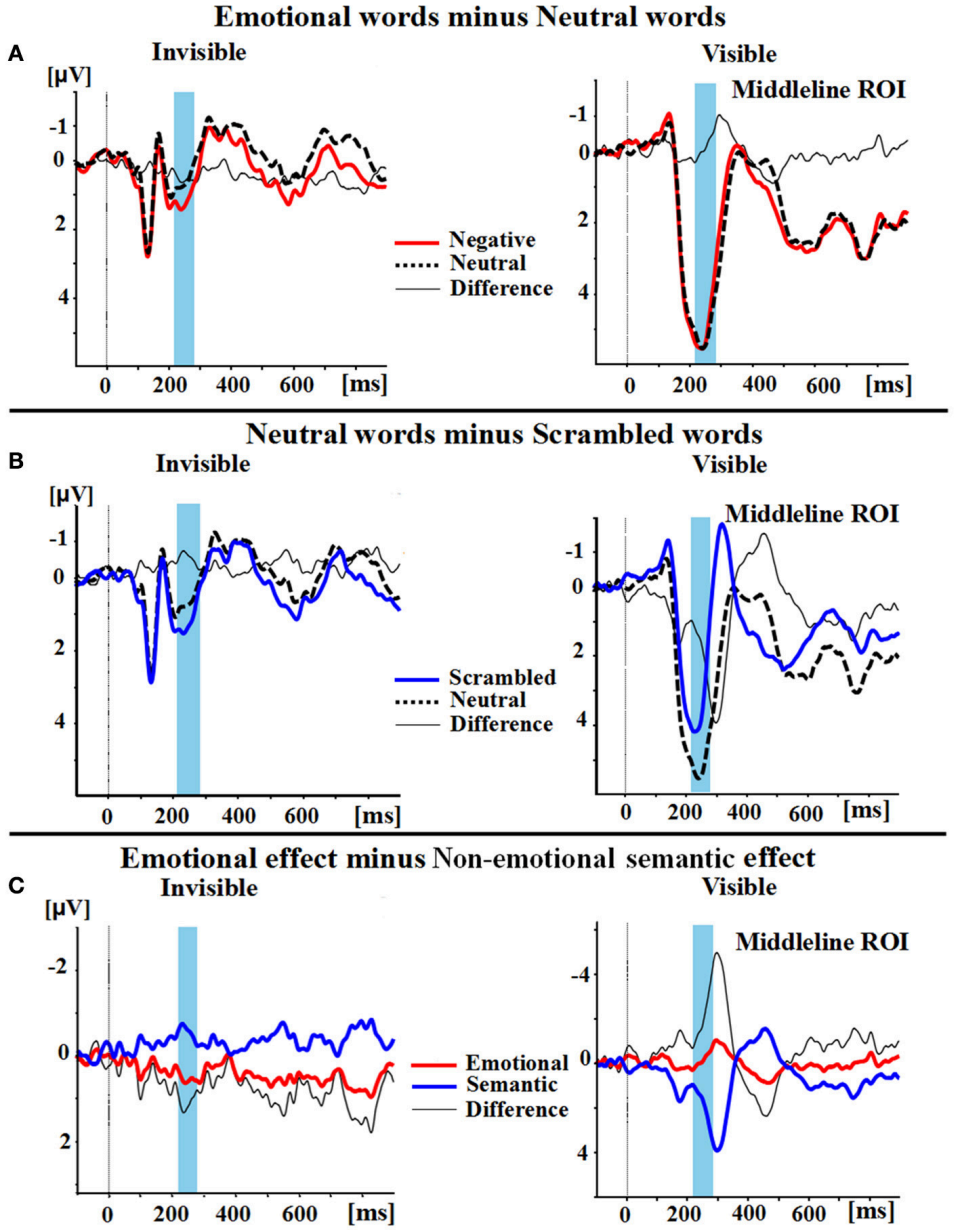
**Group Average differences in wave amplitudes from 220 to 280 ms. (A)** Emotional effect: the P2 amplitudes of negative words (red line) minus the amplitudes of neutral words (black dotted line); the difference wave is represented as a thin black line. **(B)** Semantic effect: the P2 amplitudes of neutral words (black dotted line) minus the amplitudes of scrambled words (blue line); the difference wave also is again represented as a thin black line. **(C)** The difference wave (thin black line) between the emotional effect (red line) and the semantic effect (blue line).

**Figure 3:**
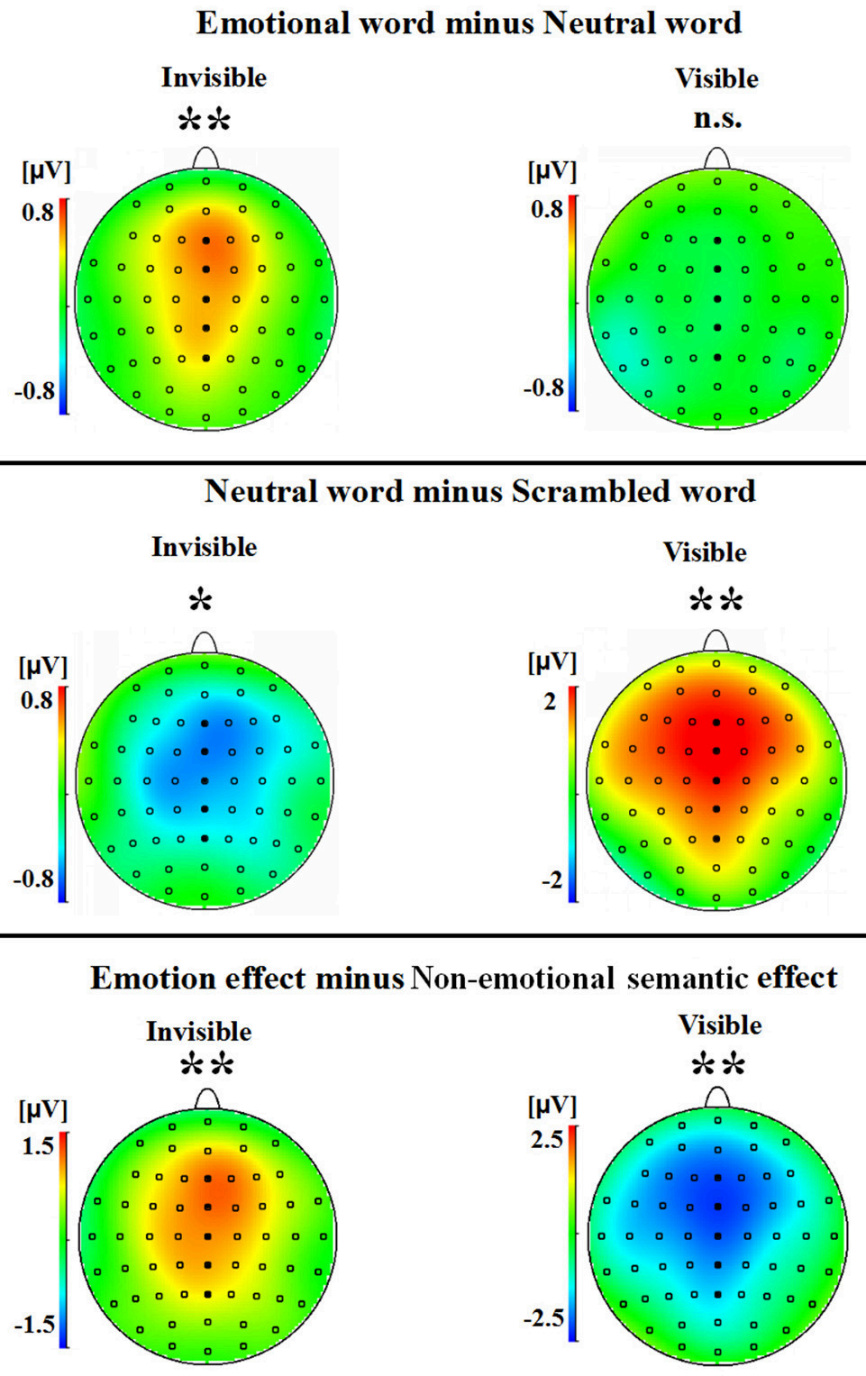
**A topographical representation of the difference waves shown in Figure 2**. The asterisks indicate statistical significance (n.s., not significant; ^*^p < 0.05, ^**^p < 0.01).

A two-way ANOVA with awareness states (visible, invisible) and word types (negative, neutral, and scrambled) showed that the interaction of awareness states and word types was significant [*F*_(2, 30)_ = 10.969, *p* < 0.01, η_*p*_^2^ = 0.422]. The main effect of awareness states was also significant [*F*_(1, 15)_ = 39.191, *p* < 0.0001, η_*p*_^2^ = 0.723], and so was the main effect of word type [*F*_(2, 30)_ = 5.646, *p* < 0.01, η_*p*_^2^ = 0.273]. Subsequent analyses revealed that P2 showed no difference between the negative words and the neutral words in the visible condition (*p* = 0.586; see Figures 2A,3). However, the negative words (1.202 ± 0.748 μV) elicited more positive P2 than the neutral words in the invisible condition (0.640 ± 0.715 μV, *p* < 0.01). The neutral words (5.164 ± 0.473 μV) elicited a larger P2 than the scrambled words (3.336 ± 0.583 μV) in the visible condition (*p* < 0.01; see Figures 2B,3). In the invisible condition, the P2 amplitudes of the scrambled words (1.189 ± 0.665 μV) were larger than those of the neutral words (0.640 ± 0.715 μV, *p* < 0.05).

A 2 × 2 ANOVA with awareness states (visible and invisible) and effects (semantic and emotional) indicated that the interaction between semantic and emotional effects was also significant [*F*_(1, 15)_ = 22.377, *p* < 0.001, η_*p*_^2^ = 0.599]. The main effect of awareness states was significant [*F*_(1, 15)_ = 7.037, *p* = 0.018, η_*p*_^2^ = 0.319]. However, the main effect of all these effects was not significant [*F*_(1, 15)_ = 1.288, *p* = 0.274, η_*p*_^2^ = 0.079]. In the visible condition, the semantic effect (1.827 ± 0.561 μV) was larger than the emotional effect (−0.125 ± 0.225 μV, *p* = 0.006). In the invisible condition, the emotional effect (0.562 ± 0.186 μV) was larger than the semantic effect (−0.549 ± 0.197 μV, *p* = 0.006; see Figures 2C,3).

## Discussion

In this research, we investigated the P2 responses to the emotional, neutral, and scrambled words in the visible and invisible conditions. Negative words elicited larger P2 amplitudes than neutral words only in the invisible condition. Neutral words elicited larger P2 amplitudes than scrambled words in the visible condition, but smaller P2 amplitudes than scrambled words in the invisible condition. The results showed that emotional processing of words can occur unconsciously, while semantic processing of words can take place both consciously and unconsciously.

The emotional effect was significant in the invisible condition, whereas this effect was not significant in the visible condition. In the visible condition, consistent with the present study, Carretié et al. (2008) found that there was no significant difference between the neutral words and negative words. In our study, participants observed the negative, neutral, and scrambled words. They found the scrambled words much easier to distinguish than the intact words. This strong categorization effect may have suppressed the emotional effect. In contrast, the emotional effect of the P2 component occurred in the invisible condition. This result was similar to previous findings. For example, Bernat et al. (2001) found that in the supraliminal condition, no significant differences in the P2 component were exhibited between unpleasant and pleasant words, while in the subliminal condition, unpleasant words evoked larger P2 amplitudes than pleasant words. Moreover, Yun et al. (2011) also found that the masked threat words elicited a larger P250 amplitude than the neutral words in PTSD (posttraumatic stress disorder) patients. Thus, the P2 component was sensitive to emotional processing in the unconscious (invisible) condition, but not in the visible condition. This result suggested that P2 might play an important role in unconscious emotional word processing.

We found a difference in the P2 component (neutral words > scrambled words) in the visible condition. This result was similar to that of previous studies (Liu et al., 2009; Chung et al., 2010). Interestingly, when the participants were unaware of the stimuli, a P2 reversal (scrambled words > neutral words) was exhibited. Moreover, the semantic effect in the invisible condition was weaker than that in the visible condition. One possibility is that a reverse mode was operating between the different types of attentional processes adopted between the visible and the invisible condition. The participants adopted a “bottom-up” attentional style in the invisible condition, yet a “top-down” style in the visible condition. Another explanation is that the observed early semantic processing effect of the P250 component was related to the inferior frontal gyrus (Liu et al., 2013), which was activated by syntactic violations in the absence of conscious awareness (Batterink and Neville, 2013; Axelrod et al., 2014). Thus, the P2 reversal effect might imply that some early semantic processing was still operating in the invisible condition, but that this effect was much weaker than that in the visible condition. Further, studies are needed to verify our interpretation of the P2 reversal effect. In short, semantic processing appears to be suppressed in the invisible condition, but not in the visible condition.

Why was the emotional effect greater than the semantic effect in the invisible condition, yet the semantic effect was stronger than the emotional effect in the visible condition? Owing to the biological value of emotional stimuli, emotional processing holds a prioritized place (Yokoyama et al., 2013). Previous studies have provided evidence of two distinct networks of emotional processing: the cortical pathway and the subcortical pathway (Whalen et al., 1998; Morris et al., 1999; Damasio et al., 2000; Adolphs, 2004; Williams et al., 2006). For example, Naccache et al. (2005) recorded the amygdala-mediated reactions of three epilepsy patients when they observed threat words and neutral words in masked and unmasked paradigms by intracranial electroencephalography (iEEG). The brain regions relating to emotional memory were also activated, such as the dorsomedial prefrontal cortex (DMPFC, Cato et al., 2004) and ventromedial prefrontal cortex (VMPFC, Kuchinke et al., 2006). The cortical pathway of emotional processing was found to be reduced or absent in CFS, while the subcortical pathway—which included the amygdala and pulvinar—was still in operation. This is because the prefrontal cortex is related to “top-down” parieto-frontal networks, which bind conscious sensory processing (Dehaene et al., 2006). Although some researches hold the notion that cortical semantic processing still occurs in the absence of awareness (Dehaene et al., 2006; Axelrod et al., 2014), in our study the degree of semantic processing was definitely impaired. Therefore, the emotional effect was greater than the semantic effect in the invisible condition, which suggested that emotional processing is less dependent on conscious awareness than on semantic processing.

We also found the main effect of the awareness state to be significant: the amplitudes of P2 in the visible condition were larger than those in the invisible condition. This result reflected the fact that the brain reactions to the word stimuli in the visible condition were stronger than those in the invisible condition. The “unconscious binding” hypothesis suggests that, in the unconscious condition, registered and attentively grouped information can be integrated (Lin and He, 2009). However, compared with conscious binding (Crick and Koch, 1990), “unconscious binding” is fragile and weak. Thus, we found a larger P2 component in the visible condition than in the invisible condition. Moreover, the main effect of the word type was significant, which reflected that the emotional and semantic processing might exist in the visible and invisible conditions.

The behavioral data showed no differences between the RTs of negative words and those of neutral words in the invisible condition; negative words were faster than neutral words and scrambled words in the visible condition. To date, ample studies have been conducted looking at the differences in the processing of emotional vs. neutral words on the behavioral level (see Jończyk, 2016). Our findings support previous studies reporting facilitative processing of negative compared to neutral words (Kousta et al., 2009; Vinson et al., 2014; Yap and Seow, 2014). However, we did not find the behavioral differences between negative words and neutral words in the invisible condition. It was probably because the behavioral data was not as sensitive as the electrophysiological data. Moreover, in our experiment the time needed (500 ms) to break the CFS was not sufficient to elicit a behavioral reaction; Yang and Yeh (2011) found that the mean RT needed to break suppression was more than 1,600 ms. Therefore, the behavioral data in our study might be insufficient to elucidate the unconscious emotional word processing.

A limitation of our research might be the participants' anticipation or some response strategies in our task. It appears to be better if the visible and invisible conditions are mixed within one block (see Yang et al., 2014). Therefore, we suggest that future studies should further investigate this line of thought.

In conclusion, our findings provide evidence that emotional words can be processed even when the stimuli are rendered invisible, which may contribute to the ongoing debate (Zimba and Blake, 1983; Dehaene et al., 2006; Jiang et al., 2007; Hesselmann et al., 2015; Ortells et al., 2016). Furthermore, emotional information is more sensitive to unconscious processing than semantic information; a semantic effect weakly occurs only in the absence of awareness. Besides, P2 plays an important role in unconscious emotional word processing. These findings appear to suggest that emotional processing remains automatic and prioritized compared with semantic processing in the unconscious condition. Since the issue is rather complex (context- and task-dependent), the debate on emotional vs. cognitive primacy remains ongoing (see Lai et al., 2012), and further studies are needed to clarify this issue.

## Author contributions

HD, QL, YL, and HL designed performed this study. HD performed the study. HD and ZZ analyzed the data. YL, HD, and WZ wrote the paper. YL and HD contributed equally to this work.

## Funding

This work was supported by the National Natural Science Foundation of China (NSFC31470997, NSFC31571153, and NSFC81171289).

### Conflict of interest statement

The authors declare that the research was conducted in the absence of any commercial or financial relationships that could be construed as a potential conflict of interest.
