# Supplementary material for: Automatic Processing of Emotional Words in the Absence of Awareness: The Critical Role of P2
Source: Front Psychol. 2017 Apr 20;8:592. doi: 10.3389/fpsyg.2017.00592 (PMC5397533; doi:10.3389/fpsyg.2017.00592)
Supplement: Supplementary file 1 [file DataSheet1.docx]

**Appendix A**

The word stimuli used in the experimental procedure.

| **Negative** | | **Neutral** | |
| --- | --- | --- | --- |
| 悲剧 | 灾害 | 风俗 | 移民 |
| 苍蝇 | 罪恶 | 法制 | 遗产 |
| 耻辱 | 罪犯 | 烈士 | 职责 |
| 敌人 | 重伤 | 宫廷 | 钢铁 |
| 棺材 | 地狱 | 规则 | 蚂蚁 |
| 火灾 | 死刑 | 廉价 | 平民 |
| 疾病 | 凶手 | 烈火 | 开支 |
| 老鼠 | 罪名 | 命运 | 船长 |
| 叛徒 | 苦难 | 契约 | 铁路 |
| 魔鬼 | 妓女 | 狮子 | 总管 |
| 太监 | 病房 | 谈判 | 激光 |
| 危机 | 创伤 | 消防 | 秘书 |
| 血迹 | 乞丐 | 心脏 | 邮票 |
| 谣言 | 细菌 | 性命 | 边境 |
| 遗体 | 错误 | 极限 | 编辑 |
